# Cognitive trajectories and their relationships with education and diets among older adults: a network-based 10-year cohort study

**DOI:** 10.3389/fnagi.2024.1498454

**Published:** 2025-01-15

**Authors:** Xuchun Wang, Yuchao Qiao, Yudong Zhang, Yu Cui, Hao Ren, Chongqi Hao, Lixia Qiu

**Affiliations:** Department of Health Statistics, School of Public Health, Shanxi Medical University, Taiyuan, Shanxi, China

**Keywords:** older adults, cognitive trajectories, network analysis, cross-lagged panel model, mediation analysis

## Abstract

**Objectives:**

Few studies have examined the underlying mechanisms of education, diets, and cognitive function in older adults. This study analyses the relationship between cognitive trajectories, education, and different dietary patterns in older adults from a network perspective, and further explores their longitudinal associations and mediation effects.

**Methods:**

Data on cognitive trajectories were derived from the Chinese Longitudinal Healthy Longevity Survey (CLHLS) between 2008 and 2018. Group-Based Trajectory Model (GBTM) was used to identify potential heterogeneity in the longitudinal changes in cognitive function. Multinomial logistic regression and network analysis were then applied to examine the relationships between different cognitive trajectories and years of education, food variety (FV), and plant-based dietary patterns. Cross-lagged panel model was used to examine the longitudinal associations between education, FV, and plant-based diet patterns. Furthermore, we constructed a mediation model based on categorical variables for cognitive trajectories to investigate the mediating effect of FV and plant-based diet index on education and cognitive.

**Results:**

A total of 2,115 older adults were included in this study, revealing three distinct cognitive function trajectories. After controlling for potential confounders, education and dietary-related variables were associated with a cognitive stable decline trajectory (OR: 0.857/0.929/1.027) and a cognitive rapid decline trajectory (OR: 0.859/0.914, 95% CI: 0.775–0.882) compared to the cognitive stable trajectory. In the education, diet, and cognition network model, overall Plant-Based Diet Index (PDI) [expected influence (EI) = 1.82] and years of education (EI = 0.54) were the most central domains. There were longitudinal associations between education, FV, and plant-based dietary patterns, which were significant only in the slow decline group. FV acting as a mediator between education and cognitive trajectories.

**Conclusion:**

Years of education are longitudinally associated with the diet of older adults in the slow cognitive decline group. Food diversity partially mediates the relationship between years of education and cognitive trajectories. Interventions targeting education and dietary behaviors may help alleviate cognitive decline in older adults.

## 1 Introduction

With the aging of society—globally—the world faces a formidable challenge. The latest census data in China reveals that the population of adults 65 years old and above has soared to 190 million, constituting 13.5% of the overall population, signifying China's formal entry into the advanced stage of population aging. It follows that, the incidence, prevalence, and mortality rates of age-related diseases also increases, especially neurodegenerative diseases such as dementia, which severely affects the health and wellbeing of older adults (Ministry of Civil Affairs, People's Republic of China, 2022). Dementia is a chronic and progressive neurological disorder marked by a progressive decline in cognitive abilities, with particularly pronounced impairments in memory, language, judgment, attention, and executive functioning. The number of persons who are 60 years of age and older with dementia in China has reached 15.07 million, accounting for 25.5% of the 49 global patient count (Rujing et al., 2021). Each year, Alzheimer's disease, the most common cause of dementia, imposes an economic burden of 167.7 billion 50 US dollars (Jia et al., 2020), exerting tremendous pressure on families and society. The future social challenges will likely increase in level and cost (Boss et al., 2015). Therefore, adopting proactive and effective strategies to prevent dementia or to identify and intervene at an early stage to delay its progression is crucial for improving the quality of life for older adults in their later years and for reducing the socio-economic burden.

Cognitive decline is a precursor to dementia, and intervention during this stage provides an opportunity to prevent dementia (Petersen et al., 1999). Therefore, timely detection of cognitive decline in older adults, identification of modifiable risk factors is crucial for preventing the onset and progression of dementia. However, cognitive decline is a dynamic process that can worsen or reverse over time (Malek-Ahmadi, 2016). Relevant research indicated that the trajectory of cognitive decline varies around the population mean trajectory, highlighting the intra-individual and inter-individual heterogeneity in cognitive function changes (Li et al., 2017; Min, 2018). Ignoring the differences in cognitive trajectories and treating all study subjects uniformly could potentially affect the effectiveness of interventions. By discussing risk factors in a stratified manner based on these trajectories, we can gain a comprehensive understanding of cognitive changes in different population groups, thereby providing targeted interventions.

Increasing evidence suggests that education and diet play crucial roles in the development of cognitive impairment. Higher education levels are associated with slower cognitive decline (Marioni et al., 2012). Education is one of the three proxies for cognitive reserve (i.e., education, occupation, leisure activities) (Nucci et al., 2012) and the only factor linked to the volume of both gray and white matter in the brain (Foubert-Samier et al., 2012), further highlighting the extensive impact of education on brain function. Healthy dietary patterns and adequate nutrient intake are associated with healthy cognition (Berendsen et al., 2017; Tangney et al., 2014; Wengreen et al., 2013; Haring et al., 2016; Smyth et al., 2015; Chen et al., 2017; Neelakantan et al., 2018). Some interventions focusing on specific dietary patterns, nutrients, and nutritional supplements for the older people have shown success, indicating that nutrition is a modifiable factor for the older people (Jennings et al., 2019; Iuliano et al., 2021; Lin et al., 2021; Nakazaki et al., 2021).

Furthermore, multiple studies have shown that individuals with higher levels of education are more likely to maintain healthier dietary patterns (Seow et al., 1998; Loughrey et al., 2017; Thorpe et al., 2019). As mentioned earlier, healthy diets and adequate nutrient intake are linked to cognitive health. Therefore, dietary patterns and nutrient intake are likely mediators in the relationship between education and cognitive function.

However, there is limited evidence on the potential mediating role of diet in the relationship between education and cognitive function. To address this knowledge gap, we assess nutritional intake and dietary patterns in older adults using food variety (FV) and plant-based dietary patterns, and evaluate educational levels using years of education. Based on multi-wave follow-up data from the Chinese Longitudinal Healthy Longevity Survey (CLHLS) from 2008 to 2018, this study investigates the longitudinal association and mediating effect between education, diet, and cognitive function changes, stratified by cognitive trajectories. The aim is to better tailor interventions to specific populations to achieve more effective outcomes.

## 2 Methods

### 2.1 Data source

The data for this study were sourced from the CLHLS, which commenced in 1998 with follow-up surveys conducted every 3–4 years. The CLHLS survey spans 23 provinces in China and includes face-to-face household interviews with people aged 65 and older. All participants provided informed consent, with family members consenting for those unable to sign themselves (Zhang and Liu, 2007). Detailed information on the survey's sampling procedure and data quality is available in previous studies (Zeng et al., 2017). The survey has received approval from the Biomedical Ethics Committee of Peking University (IRB00001052-13074). Our team was granted data usage rights from the Peking University Open Research Data Platform.

### 2.2 Participants

This study used data from four waves of the CLHLS conducted between 2008 and 2018. Participants were older adults who completed cognitive function assessments in all four surveys. Samples missing dietary information or covariates at baseline and follow-ups were excluded, resulting in 2,115 participants. The selection process is shown in Supplementary Figure S1.

### 2.3 Food variety measurement

Food variety (FV) is a key factor in dietary quality and nutritional adequacy (Kennedy, 2009), ensuring a rich supply of all food groups as well as a wide range of macro and micronutrients (Kennedy et al., 2011). In this study, the Food Variety Score (FVS) was used to assess the food variety consumed by older adults from 2008 to 2018. The FVS was calculated based on the frequency of consumption of 13 specific food types included in the CLHLS questionnaire. These food types are: fresh fruits, fresh vegetables, meat, fish, eggs, legumes, preserved vegetables, sugar, garlic, dairy products, nuts, mushrooms, and tea. For fruit and vegetable consumption, respondents selected from four frequency options: daily, often, occasionally, or almost never. A score of 1 was assigned for “daily” or “often” consumption, while a score of 0 was assigned for “occasionally” or “rarely.” For the remaining 11 food items, consumption frequency was categorized into five options: daily, weekly, monthly, occasionally, and almost never. Again, a score of 1 was assigned for “daily” or “weekly” consumption, and a score of 0 was assigned for “monthly,” “occasionally,” or “almost never.” Each participant's final FVS was obtained by summing the scores across all food categories, yielding a range from 0 to 13. A higher score indicates a more diverse diet (Zhang et al., 2022).

### 2.4 Calculation of plant-based diet indices

Instead of focusing on individual foods or nutrients, overall dietary patterns consider the combined effects of multiple foods and nutrients. Given that plant-based diets align well with Asian dietary habits, this study used Plant-Based Diet Indices to assess older adults' dietary patterns. The dietary information for calculating the plant-based diet indices was primarily obtained through a simplified Food Frequency Questionnaire (FFQ), and these data were used to compute the plant-based diet scores. Initially, based on the nature of the foods, 16 common foods from the Chinese daily diet were grouped into three categories [in contrast to food diversity, the plant-based dietary pattern also considers the intake of grains (divided into whole grains and refined grains) and edible oil]: (1) healthful plant-based foods (whole grains, fresh fruits, fresh vegetables, legumes, garlic, vegetable oils, nuts, and tea), (2) unhealthful plant-based foods [refined grains, preserved vegetables, and sugar (white granulated sugar or candies)], and (3) animal-based foods (animal fat, eggs, fish and aquatic products, meat, and milk and dairy products). The questionnaire offered five response options for legumes, garlic, nuts, tea, salted preserved vegetables, sugar, eggs, fish, meat, and milk: “almost every day,” “≥1 time per week,” “≥1 time per month,” “occasionally,” and “rarely or never.” For whole grains, refined grains, vegetable oils, and animal fats, participants could respond with either “yes” or “no.” For fruits and fresh vegetables, the available choices were “almost every day,” “quite often,” “occasionally,” or “rarely or never.”

Using self-reported dietary frequencies, we calculated three plant-based diet indices: the overall Plant-Based Diet Index (PDI), the healthful Plant-Based Diet Index (hPDI), and the unhealthful Plant-Based Diet Index (uPDI). The consumption of each food was taken into account when calculating these indices. In line with previous studies (Satija et al., 2016, 2019, 2017), each food was assigned a score ranging from 1 to 5, with the specific focus of each index varying slightly. For the PDI, positive scores were assigned to plant-based food groups (with 1 indicating the least frequent consumption and 5 the most frequent), while animal-based food groups received reverse scores (fivee for the least frequent and one for the most frequent consumption). For the hPDI, healthful plant-based foods were scored positively, whereas unhealthful plant-based foods and animal-based foods were given reverse scores. In the case of the uPDI, positive scores were assigned to unhealthful plant-based foods, while both healthful plant-based foods and animal-based foods received reverse scores. The PDI, hPDI, and uPDI scores were calculated by summing the scores for each of the 16 food groups, with a theoretical range from 16 to 80.

### 2.5 Cognitive and education assessment

The CLHLS measured global cognitive function with the Chinese version of Mini-Mental State Examination (MMSE). The scale comprises 24 items, yielding a maximum score of 30 points, where higher scores indicate better cognitive function. It focuses on five primary domains: general ability, responsiveness, attention and calculation ability, recollection, language comprehension, and self-coordination. The validity and reliability of the Chinese MMSE have been verified (Chan et al., 2002; Lök et al., 2019).

The assessment of education level in the elderly population was primarily based on self-reported years of schooling.

### 2.6 Covariates

This study included demographic characteristics (gender, age, region, marital status, living conditions), socioeconomic status (Household income, wealth status, and health insurance coverage), and lifestyle factors (smoking, drinking, Exercise and BMI.) as covariates (Supplementary material).

### 2.7 Statistical analysis

Categorical variables were analyzed using frequencies (%) and continuous variables using means (SD). To study the cognitive trajectories of older adults, we employed a Group-Based Trajectory Model (GBTM) (Nagin, 1999). Additionally, a Mixed Graph Model (MGM) (Haslbeck and Waldorp, 2015) was utilized to construct a network model to explore the interconnections between education, dietary patterns, and cognitive trajectories. The Expected Influence (EI) metric was used to assess the core domains within the education, diet, and cognition network, with higher EI values indicating a stronger influence of a node on other nodes within the network. Detailed descriptions of the GBTM and network analysis methods are provided in the Supplementary material. To enhance the reliability of the network results, we conducted two sensitivity network analyses while controlling for potential confounding factors.

To examine the longitudinal associations between baseline education and follow-up FV, PDI, hPDI, and uPDI, we employed a cross-lagged Structural Equation Model (SEM) (Hamaker et al., 2015). This model simultaneously estimates multiple regression equations and assesses the directionality of associations while adjusting for potential confounders.

Finally, we analyzed the mediating mechanisms between education, diet, and cognitive trajectories using mediation analysis methods suited for categorical dependent variables. We adjusted the model linking the outcome variable (Y), mediator (M), and independent variable (X) from linear regression to cumulative logistic regression to accommodate the multicategorical nature of our outcome data. The mediating effect was tested using the product of coefficients method. Detailed descriptions of the methodology can be found in the referenced literature (Iacobucci, 2012).

All statistical analyses were performed using R 4.4.0 (R Core Team, Vienna, Austria). Two-sided *p*-values < 0.05 were considered statistically significant.

## 3 Results

### 3.1 Basic characteristics of different cognitive trajectories

Based on the selection criteria in Supplementary Table S2 and the cognitive trajectory curves in Supplementary Figure S3, we ultimately identified three cognitive trajectories: “High Stability” with 1,102 individuals (52.1%), “Slow Decline” with 710 individuals (33.3%), and “Rapid Decline” with 303 individuals (14.3%). Table 1 displayed the basic characteristics of the population in each cognitive trajectory group. In comparison to the other two cognitive trajectories, older adults in the “Rapid Decline” group were likely to be older, female, unmarried, not wealthy, never smokers, never drinkers, never exercisers, underweight, have lower years of education, have more children, lower PDI, hPDI, FV, and higher uPDI. Details on the whole sample can be found in Supplementary Table S1 and Supplementary Figure S2.

**Table 1:** Baseline characteristics of the sample by the different trajectory groups.

**Factors**	**Level**	**High stability**	**Slow decline**	**Rapid decline**	***P***
		**1102**	**710**	**303**	
Gender (%)	Female	450 (40.8)	447 (63.0)	210 (69.3)	< 0.001
	Male	652 (59.2)	263 (37.0)	93 (30.7)	
Ethnic (%)	Others	71 (6.4)	41 (5.8)	28 (9.2)	0.120
	Han nationality	1,031 (93.6)	669 (94.2)	275 (90.8)	
Rural (%)	Rural	987 (89.6)	668 (94.1)	285 (94.1)	0.001
	Urban	115 (10.4)	42 (5.9)	18 (5.9)	
Marital (%)	Others	339 (30.8)	338 (47.6)	186 (61.4)	< 0.001
	Married	763 (69.2)	372 (52.4)	117 (38.6)	
Econ state (%)	No	940 (85.3)	643 (90.6)	271 (89.4)	0.002
	Yes	162 (14.7)	67 (9.4)	32 (10.6)	
Medical Insurance (%)	No	931 (84.5)	609 (85.8)	261 (86.1)	0.657
	Yes	171 (15.5)	101 (14.2)	42 (13.9)	
Co_residence (%)	No	966 (87.7)	587 (82.7)	240 (79.2)	< 0.001
	Yes	136 (12.3)	123 (17.3)	63 (20.8)	
Smoke (%)	Never	620 (56.3)	488 (68.7)	233 (76.9)	< 0.001
	Formerly	176 (16.0)	88 (12.4)	31 (10.2)	
	Current	306 (27.8)	134 (18.9)	39 (12.9)	
Drink (%)	Never	687 (62.3)	464 (65.4)	222 (73.3)	0.001
	Formerly	136 (12.3)	103 (14.5)	32 (10.6)	
	Current	279 (25.3)	143 (20.1)	49 (16.2)	
Exercise (%)	Never	582 (52.8)	417 (58.7)	191 (63.0)	< 0.001
	Formerly	86 (7.8)	66 (9.3)	32 (10.6)	
	Current	434 (39.4)	227 (32.0)	80 (26.4)	
BMI (%)	< 18.5	188 (17.1)	173 (24.4)	90 (29.7)	< 0.001
	18.5–24.0	645 (58.5)	396 (55.8)	164 (54.1)	
	24.0–28.0	211 (19.1)	115 (16.2)	39 (12.9)	
	≥28.0	58 (5.3)	26 (3.7)	10 (3.3)	
Age [mean (SD)]	–	72.41 (6.47)	75.79 (6.90)	82.17 (8.34)	< 0.001
Education [mean (SD)]	–	4.19 (4.00)	1.64 (2.72)	1.10 (2.49)	< 0.001
Children number [mean (SD)]	–	4.03 (1.75)	4.52 (1.96)	4.78 (2.26)	< 0.001
Income [mean (SD)]	–	19546.51 (23,507.46)	18003.34 (24,202.16)	19481.10 (26,450.77)	0.389
mmse [mean (SD)]	–	28.71 (1.38)	24.52 (3.82)	11.78 (8.51)	< 0.001
PDI [mean (SD)]	–	50.83 (6.20)	50.78 (6.10)	49.75 (6.43)	0.022
hPDI [mean (SD)]	–	48.45 (5.68)	48.05 (5.70)	47.65 (5.55)	0.065
uPDI [mean (SD)]	–	48.79 (5.30)	49.81 (5.50)	49.41 (5.55)	< 0.001
FV [mean (SD)]	–	6.03 (2.51)	5.30 (2.35)	5.01 (2.42)	< 0.001

**Figure 1:**
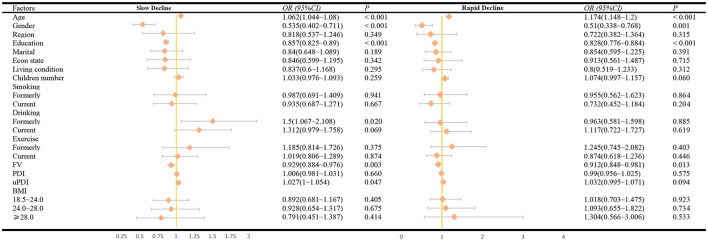
The factors associated with cognitive trajectories.

In the multinomial logistic regression analysis with the “High Stability” group as the reference, age (OR: 1.063/1.174), gender (OR: 0.532/0.506), years of education (OR: 0.857/0.829), and FV (OR: 0.929/0.914) were common determinants for both the “Slow Decline” and “Rapid Decline” groups. uPDI (OR: 1.501) and formerly drinking (OR: 1.027) were determinants for the “Slow Decline” group. See Figure 1 for details.

### 3.2 Network analysis results

**Figure 2:**
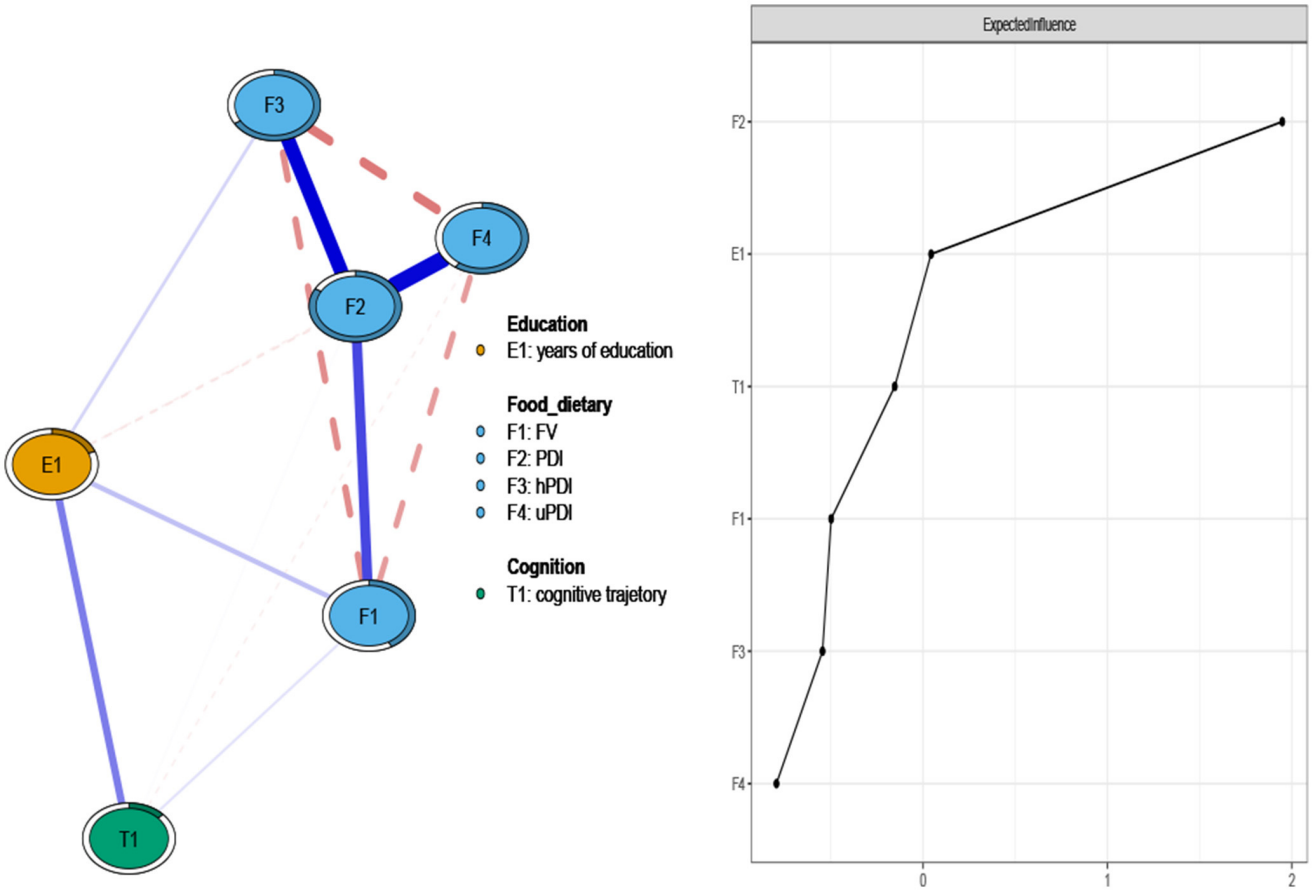
Network analysis of education, diets, and cognition in older adults (primary network analysis). The cognitive trajectory groups in our study are defined as follows: high stability group = 3, slow decline group = 2, and rapid decline group = 1. By default, higher values indicate more stable cognitive function.

Figure 2 illustrated the network structure and centrality of education, diet, and cognitive function in older adults (primary network model). PDI (EI = 1.85) and education (EI = 0.54) emerged as the most central domains within the education, diet, and cognition model. Positive correlations were observed between cognition and education (edge weight > 0), as well as between both education and cognition with FV (edge weight > 0). In the sensitivity network analyses presented in Supplementary Figures S4, S5, even after adjusting for covariates affecting the relationships among education, diet, and cognition, the central domains remained consistent with those found in the primary network analysis. The stability and accuracy of the primary network analysis are supported by Supplementary Figure S6, which shows a cs-coefficient for EI of 0.75, indicating stable and accurate centrality values. Supplementary Figure S7 demonstrates the reliability and stability of the edges through the bootstrap range of the estimated edge weights' 95% confidence intervals. Supplementary Figures S8, S9 highlight significant differences in EI and edge weights within the network model.

### 3.3 Association between cognitive trajectories and education years and diet

Supplementary Table S3 presents the unadjusted and adjusted estimates of the association between years of education, diet-related scores, and cognitive function from the multinomial logistic regression analysis. In Model 1, while without adjusting for any covariates, individuals in the “Slow Decline” group had a reduced chance of cognitive decline if they had higher FV scores and more years of education, as well as lower uPDI scores, compared to those in the “High Stability” group. Similarly, individuals in the “Rapid Decline Class” who had higher FV scores, PDI scores, hPDI scores, and more years of education had a reduced chance of cognitive decline compared to those in the “High Stability” group. In Model 2 and 3, after controlling for age, gender, region, marital, econ state, living conditions, children number (Model 2), Smoke, Drink, Exercise and BMI (Model 3), the associations between FV, years of education, and cognitive trajectories remained unchanged for both the “Slow Decline” and the “Rapid Decline” compared to the “High Stability”.

### 3.4 Longitudinal association between education, diet and cognition

**Figure 3:**
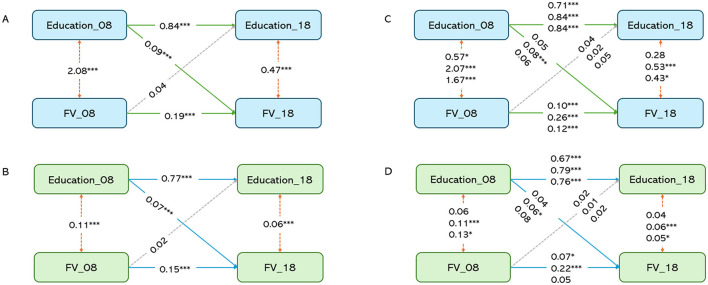
Cross lagged model diagram between education and FV. **(A)** Unadjusted and stratified original model; **(B)** covariate-adjusted model; **(C)** coarse model stratified according to trajectory group; **(D)** covariate-adjusted and trajectory group stratified model. *P < 0.05, **P < 0.01, ***P < 0.001.

Figure 3 illustrated the path relationship between years of education and FV among the older adults. It is evident that baseline years of education were associated with baseline FV (r1 = 2.08, *P* < 0.001), baseline years of education were associated with follow-up years of education (β1 = 0.84, *P* < 0.001), and baseline FV was associated with follow-up FV (β2 = 0.19, *P* < 0.001), indicating a certain level of stability in education years and FV over 10 years. After adjusting for gender, age, marital, econ state, smoking, drinking, and exercise, the path coefficient from baseline FV to follow-up years of education was not statistically significant (ρ1 = 0.003, *P* > 0.05), while the path coefficient from baseline years of education to follow-up FV was statistically significant (ρ2 = 0.009, *P* < 0.001). These results suggest a unidirectional causal relationship between years of education and FV.

Further stratification based on 10-year cognitive trajectories revealed that after adjusting for covariates, the association between baseline years of education and follow-up FV was significant only in the “Stable Decline”. This finding indicates that the influence of education on FV is primarily observed among older people experiencing a slow decline cognitive trajectory.

Based on the above analytical steps, we further examined the longitudinal associations between years of education and three dietary patterns (PDI, hPDI, and uPDI) as well as cognitive. Similar to the findings for FV, years of education showed significant temporal associations with PDI, hPDI, and cognition in both the overall unstratified model and the stratified slow decline cognitive trajectory. However, the association with uPDI was not significant, indicating no temporal relationship between baseline years of education and follow-up uPDI. Detailed results can be found in Supplementary Figures S10–S13.

### 3.5 Mediating effect

**Figure 4:**
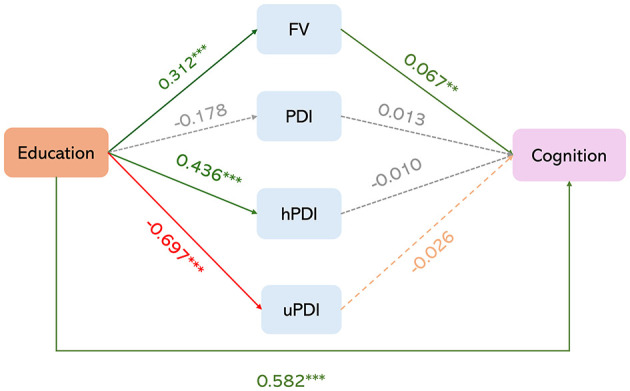
Mediation analysis of education and cognitive function changes mediated by FV and plant-based dietary patterns (adjusted covariates). *P < 0.05, **P < 0.01, ***P < 0.001.

Based on the above research results, to verify the mediating effect of diet on the relationship between years of education and changes in cognitive function in the older adults, we further conducted a mediation analysis with baseline years of education as the independent variable, follow-up FV, PDI, hPDI, and uPDI as mediating variables, and cognitive trajectories as the dependent variable. The results, shown in Table 2 and Figure 4, indicated that FV was the only significant mediating factor before and after controlling for covariates. The results demonstrated that both the direct effect of years of education and the indirect effect of FV on cognitive function were significant (95% CI did not include 0). The mediation effect explained 3.5% [0.021/(0.021 + 0.582)] of the total variance. These findings suggest that FV mediates the impact of years of education on changes in cognitive function.

**Table 2:** Mediating effects of FV and plant-based dietary patterns on the relationship between education and cognitive function changes.

	**Model1**	**Model2**	**Model3**
**Effect**	**Estimate (95% CI)**	**Estimate (95% CI)**	**Estimate (95% CI)**
Direct effect	0.896 (0.778–1.013)	0.582 (0.454 to −0.709)	0.582 (0.453–0.710)
Indirect effect (FV)	0.038 (0.012–0.066)	0.024 (0.006–0.045)	0.021 (0.006 to −0.040)
Indirect effect (PDI)	0.007 (−0.002 to 0.021)	−0.001 (−0.010 to 0.006)	−0.002 (−0.014 to 0.006)
Indirect effect (hPDI)	−0.010 (−0.030 to 0.009)	−0.006 (−0.022 to 0.008)	−0.004 (−0.019 to 0.008)
Indirect effect (uPDI)	0.020 (0.004–0.039)	0.018 (< 0.001–0.040)	0.018 (−0.0003 to 0.039)
Total effect	0.934 (0.813 to −1.054)	0.605 (0.477–0.735)	0.603 (0.473–0.732)

Model 1: Crude Model; Model 2, adjusted for age, gender, region, marital, econ state, living conditions, children number; Model 3, model 2 + smoke, drink, exercise and BMI.

## 4 Discussion

This longitudinal study adds to the limited research on the changes in cognitive function over time in older adults and helps us understand the factors influencing different cognitive trajectories in individuals aged 65 and above. Additionally, this study further elucidates the mediating role of diet between educational attainment and cognitive function. The results indicated that there were three distinct trajectories of cognitive function over a 10-year period in older adults: high stability, slow decline, and rapid decline. Among these, the high stability group comprises the largest proportion of participants. These findings are consistent with the results of Tampubolon (2015), Min (2018), and Tu et al. (2020), suggesting that cognitive function remains stable for the majority of participants during aging.

Additionally, the analysis of related factors in this study indicated that characteristics such as education, FV, age, gender, drinking habits, and uPDI were associated with cognitive decline. These findings are consistent with the results of Tu et al. (2020) and Wu et al. (2019), which also validated the associations between age, gender, educational attainment, diet quality, and cognitive function in older adults. It demonstrates that higher educational attainment, being male, and better diet quality can reduce the risk of cognitive decline.

Moreover, our study revealed an interesting phenomenon: overall, baseline education level was significantly associated with FV, PDI, hPDI, and cognitive function at the 2018 follow-up, which is consistent with most previous research conclusions. Higher levels of education predict that individuals are more likely to maintain healthier dietary behaviors (Kant, 2004; Harrington et al., 2014; Mishra et al., 2006; Tabung et al., 2016) or improve their dietary behaviors over time (Harrington et al., 2014; Arabshahi et al., 2011); higher education levels tend to be associated with slower cognitive decline (Marioni et al., 2012). However, stratified analyses revealed that these significant associations did not apply to all older adults. For instance, the longitudinal associations between education and FV, PDI, and hPDI were observed only in the group with slow cognitive decline, while the longitudinal association between education and cognitive function was significant only in the stable group. Considering the differences in mean years of education among the cognitive trajectory groups, the high stability group had generally higher educational attainment compared to the slow decline and rapid decline groups, with the rapid decline group having the lowest educational levels. To our knowledge, the influence of education on dietary behavior is likely related to the ability to acquire, understand, and implement knowledge about ideal dietary behaviors. Individuals with low educational attainment have consistently shown lower overall dietary quality levels, possibly due to a lack of nutrition knowledge, cooking skills, or the ability to use preventive information. Those who have received a certain level of schooling are capable of acquiring these skills, thereby meeting the essential requirements for adopting healthy dietary behaviors and improving their diet. This group, with a certain educational foundation and situated at the boundary of having or lacking the ability to change dietary behaviors, is likely to exhibit characteristics of slow cognitive decline. The reason why the impact of education on cognition exists as a longitudinal association only in the high stability cognitive function group might be that this group has relatively higher education levels, while the education levels of the other two groups are too low to influence cortical functions and other behaviors mediating cognitive function, or it may be that their relationship is offset by other factors. These conclusions require further in-depth research and verification, but they also provide a new research perspective for related fields.

Finally, the results of our study demonstrated that FV has a significant mediating effect on the relationship between years of education and changes in cognitive function. The above findings are consistent with several studies. Firstly, individuals with higher education levels are more likely to maintain a healthy diet. Secondly, healthy dietary patterns and adequate variety in food intake can improve cognitive function by affecting metabolism (Galbete et al., 2018), inflammation (Azadbakht et al., 2005; Miller et al., 1992), and microvascular function (Yaffe et al., 2014). This suggests that FV may partially mediate the relationship between education and cognitive function.

This study has several strengths. First, we are the first to demonstrate the bidirectional association between educational attainment, dietary patterns, and cognitive function among individuals with different cognitive function trajectories, using data from a nationally representative long-term cohort. This provides strong support for the associations described in our study. Second, we utilized multi-time point cohort data spanning 10 years to explore longitudinal associations, thereby gaining a profound understanding of the relationships between education, diet, and cognitive function. This will provide guidance for interventions aimed at maintaining cognitive function in the elderly. Third, we assessed the potential mechanisms underlying this association, showing that the relationship between educational attainment and cognitive function is partially mediated by food variety, which may further enhance health interventions.

Despite its contributions, this study has several limitations. First, due to resource limitations, cognitive function was assessed solely using the MMSE scale. As a rapid screening tool, the MMSE may not capture the full complexity of cognitive function, potentially limiting the precision of cognitive assessments. However, MMSE has been widely validated as a reliable instrument for large-scale population studies. Second, both education level and dietary information were self-reported by the participants, which may introduce potential biases. Furthermore, the lack of more detailed and representative assessment metrics represents an inherent limitation of this study. More comprehensive evaluations are crucial for future in-depth research. Nevertheless, the measurement methods employed in this study have been validated as effective in previous studies (Zhang et al., 2022; Qi et al., 2023; Yi and Vaupel, 2010). Third, our study examined only one potential mediating mechanism through food variety (FV). While the mediating effect of FV was statistically significant, it accounted for only a small portion of the total effect, indicating that other unexamined mediators likely exist and require further exploration. Fourth, some participants were excluded due to missing data or loss to follow-up, which may introduce selection bias and affect the representativeness of the findings. Future research will involve combining multiple representative cohort datasets to better understand the relationships between education, diet, and cognition. Additionally, when feasible, we aim to construct cohort datasets for related research topics to conduct more accurate and representative assessments of population cognition, educational levels, and dietary patterns, thus enhancing the reliability of the associations observed. Finally, regarding the dietary assessment in this study, the lack of information on the proportions of various food components represents a key area for future research. Investigating the role of food component proportions in the identified associations will be an important direction for subsequent studies.

## 5 Conclusion

In conclusion, this study indicated that there was a longitudinal association between educational attainment and cognitive function over time among older adults in China, and this prospective education-cognition relationship was partially mediated by FV. Given these findings, healthcare providers can focus more on older adults with lower educational levels and inadequate food variety intake, taking effective measures to mitigate cognitive function disparities caused by low educational attainment and addressing cognitive decline by enriching food variety.

## Data Availability

Publicly available datasets were analyzed in this study. This data can be found here: https://opendata.pku.edu.cn/dataverse/CHADS (accessed on 27 April 2024).
