# Supplementary material for: Cognitive trajectories and their relationships with education and diets among older adults: a network-based 10-year cohort study
Source: Front Aging Neurosci. 2025 Jan 15;16:1498454. doi: 10.3389/fnagi.2024.1498454 (PMC11775158; doi:10.3389/fnagi.2024.1498454)
Supplement: Supplementary file 1 [file Data_Sheet_1.docx]

Supplementary Material

# Methods

## Participants


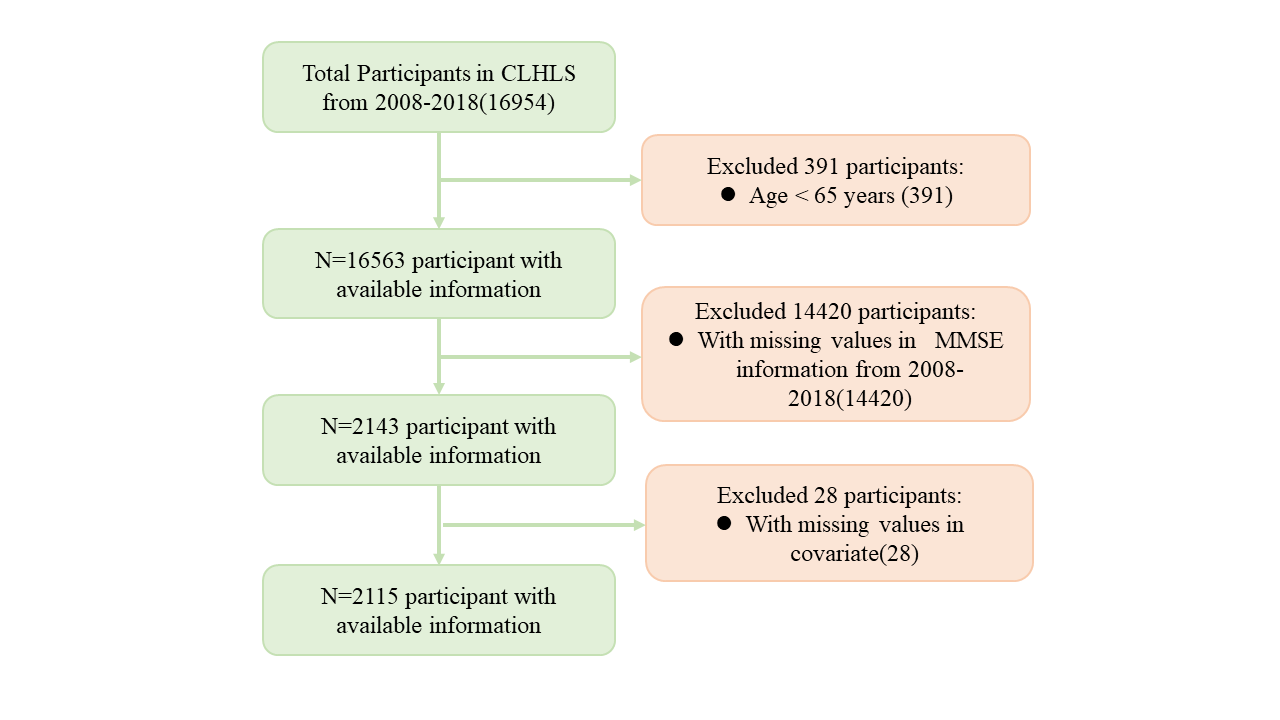


**Supplementary Figure 1.** Flowchart of Participant Screening Flowchart

## Covariates

This study included demographic characteristics, socioeconomic status, and lifestyle factors as covariates. Demographic characteristics encompassed gender (female or male), age, region (urban or rural), marital status (married/partnered or other), and living conditions (living alone or not). Household income, wealth status, and health insurance coverage were classified as socioeconomic status-related characteristics. For lifestyle factors, we considered the participants' smoking status (current, former, or never), drinking status (current, former, or never), Exercise (current, former, or never), and BMI. BMI was categorized into four groups: underweight (BMI < 18.5 kg/m²), normal weight (18.5 kg/m² ≤ BMI < 24.0 kg/m²), overweight (24 kg/m² ≤ BMI < 28.0 kg/m²), and obese (BMI ≥ 28.0 kg/m²).

## GBTM

GBTMs, also known as latent class growth models, are used to model and analyze longitudinal data with population heterogeneity. Semi-parametric mixture models were fitted to the longitudinal data using maximum likelihood methods to approximate the unknown trajectories of the population members. The time metric was the years since baseline (2008-2018), and the outcome was the MMSE score assessed over 1-4 waves. Following general recommendations, we fitted models with 1 to 5 trajectories, each with 1 to 3 polynomial functions, and identified the most meaningful model (P<0.05). The optimal model was determined based on the AIC, BIC, average posterior probability (AvePP), and correct classification probability (OCC) for each group. The specific selection criteria were as follows: ① smallest absolute AIC and BIC scores; ② average AvePP>0.7 for each trajectory group; ③ each trajectory group comprised >5% of the sample.

## Network Analysis

Network analysis is a novel method that has gained widespread use in recent years, allowing for the visualization of relationships among individual symptoms or factors. In a network model, each cognitive trajectory and related factor is represented as a node, with the associations between nodes (partial correlation coefficients) depicted as edges. In our network analysis, we employed a mixed graphical model to construct the network structure of cognitive trajectories, education, and diet. Blue edges indicate positive associations between nodes, while pink dashed edges indicate negative associations. The thickness of the edges represents the strength of the associations.

To enhance the clarity of the model, we applied the graphical least absolute shrinkage and selection operator (LASSO) regression to sparsify the network structure, retaining more realistic node connections. We used the Extended Bayesian Information Criterion (EBIC) to select the network model, ultimately retaining edges with stronger weights.

The centrality of the network model, or the importance of nodes within the network, was evaluated using the Expected Influence (EI) metric. This metric is commonly used for assessing the centrality of nodes in a network model that includes both positive and negative edges. An EI value greater than zero for a specific node indicates that the absence of this node would lead to a reduction in symptoms within the model, and vice versa. Higher EI values signify stronger associations with other nodes and a greater influence on the entire network model. The stability of the network structure and EI values was validated using a bootstrap procedure.

# Results

## Description of the study sample

Table S1 and Figure S2 present the baseline characteristics of the participants in this study. Among the 2,115 participants included in the analysis, the average age was 74.94 years, with 1,008 men (47.7%). The majority were married (59.2%) and rural residents (91.7%). The mean years of education were 2.89 years, and the mean scores for FV, PDI, hPDI, uPDI, and MMSE were 5.64, 50.66, 48.20, 49.22, and 27.53, respectively. Except for uPDI, the differences in years of education, FV, PDI, hPDI, and MMSE scores between genders were statistically significant, with males scoring higher than females. The mean MMSE scores across the four waves of the survey were 27.53, 27.43, 27.13, and 24.88, respectively. The differences in cognitive scores between 2014 and 2018 compared to 2011 were statistically significant, as was the difference between 2014 and 2018. Overall, there was a declining trend in cognitive function scores among older adults over the 10-year period.

**Supplementary Table 1.** Baseline characteristics of the total sample

| Factors | level | Overall |
| --- | --- | --- |
| Gender (%) | Female | 1107 (52.3) |
|  | Male | 1008 (47.7) |
| Ethnic (%) | Others | 140 (6.6) |
|  | Han nationality | 1975 (93.4) |
| Birth_place (%) | Rural | 1940 (91.7) |
|  | Urban | 175 (8.3) |
| Marital (%) | Others | 863 (40.8) |
|  | married and living with spouse | 1252 (59.2) |
| Econ state (%) | No | 1854 (87.7) |
|  | Yes | 261 (12.3) |
| Medical Insur (%) | No | 1801 (85.2) |
|  | Yes | 314 (14.8) |
| Co_residence | No | 1793 (84.8) |
|  | Yes | 322 (15.2) |
| Smoke (%) | Never | 1341 (63.4) |
|  | Formerly | 295 (13.9) |
|  | Current | 479 (22.6) |
| Drink (%) | Never | 1373 (64.9) |
|  | Formerly | 271 (12.8) |
|  | Current | 471 (22.3) |
| Exercise (%) | Never | 1190 (56.3) |
|  | Formerly | 184 (8.7) |
|  | Current | 741 (35.0) |
| BMI (%) | <18.5 | 451 (21.3) |
|  | 18.5~24.0 | 1205 (57.0) |
|  | 24.0~28.0 | 365 (17.3) |
|  | ≥28.0 | 94 (4.4) |
| Age (mean (SD)) | - | 74.94 (7.67) |
| Education (mean (SD)) | - | 2.89 (3.69) |
| Children Number (mean (SD)) | - | 4.30 (1.92) |
| MMSE (mean (SD)) | - | 27.53 (3.68) |
| Income (mean (SD)) | - | 19018.88 (24179.90) |
| PDI (mean (SD)) | - | 50.66 (6.21) |
| hPDI (mean (SD)) | - | 48.20 (5.67) |
| uPDI (mean (SD)) | - | 49.22 (5.42) |
| FV (mean (SD)) | - | 5.64 (2.48) |
| FI (mean (SD)) | - | 0.11 (0.05) |
| BMI (mean (SD)) | - | 21.45 (3.70) |


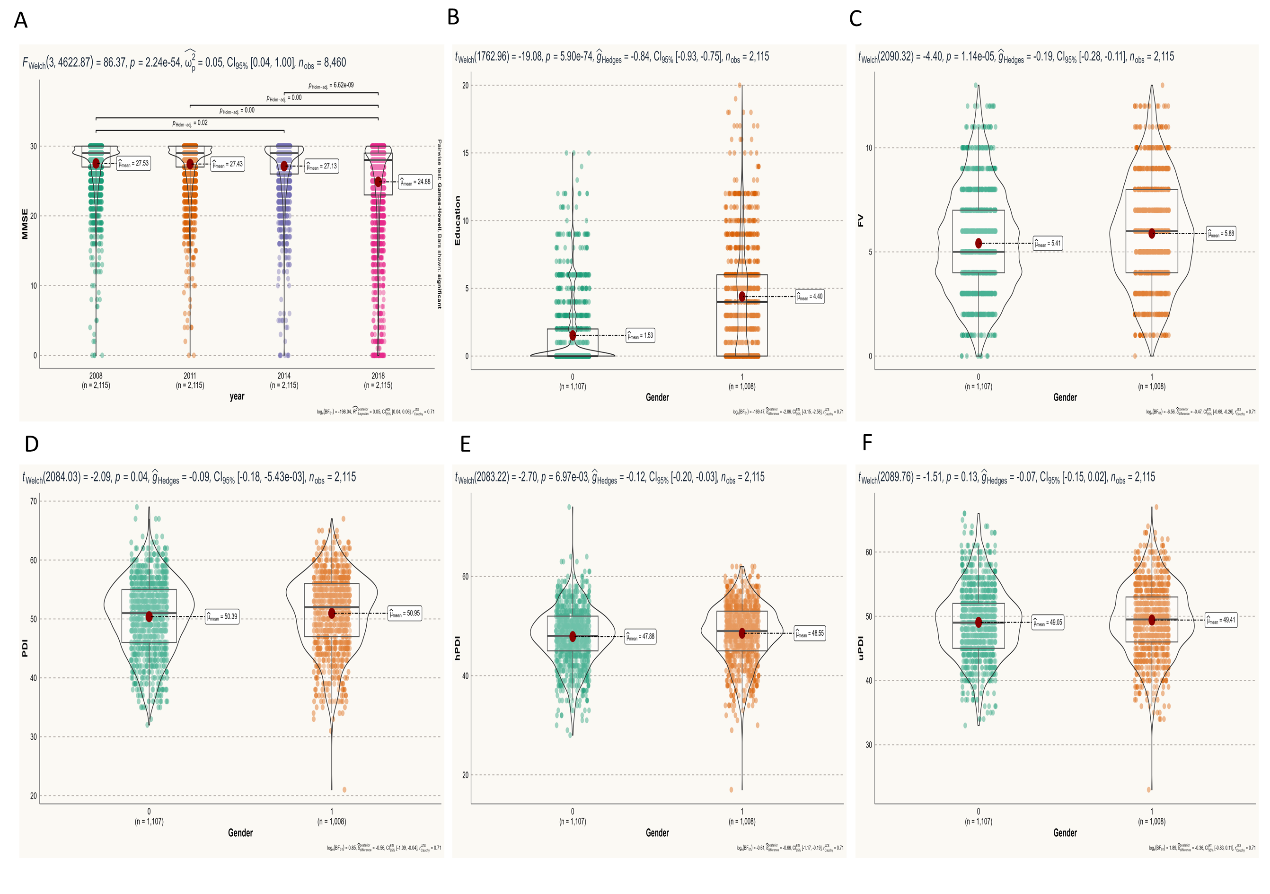


**Supplementary Figure 2.** Distribution of cognitive function, diet, and education of study subjects

## Cognitive trajectory models

To further investigate the trajectories of cognitive function in older adults and distinguish subpopulations with different trends, we utilised the GBTM model for latent class trajectory analysis on longitudinal multi-wave data. As shown in Table S2, the values of AIC, BIC gradually decreased with the increase in the number of classes. The entropy values of all models exceeded the critical threshold of 0.70, and the minimum OCC values for different trajectories were greater than 5, indicating a good model fit. The LMR and BLRT significance tests showed significant differences between classes. However, when the model was partitioned into five classes, the tiniest class represented merely approximately 6% of the entire population, which constituted an insufficient sample size. Therefore, the model ultimately selected four classes as the potential number of classes, and we plotted the trajectory change curves. As seen in Figure S3, Trajectories 2 and 4 were both stable. Consequently, we merged the populations of Trajectories 2 and 4 into one class for later cognitive function trajectory categorisation. After careful consideration of all the considerations, we ultimately extracted three classes as the final number of potential classes. Based

on the conditional means across dimensions for each latent class (Figure 4), we named the merged group as the "High Stability Class" with 1,102 individuals (52.1%), Trajectory 1 as the "Stable Decline Class" with 710 individuals (33.3%), and Trajectory 3 as the "Rapid Decline Class" with 303 individuals (14.3%).

**Supplementary Table 2.** Fit indices of each latent class model

| model | G1 | G2 | G3 | G4 | G5 | OCC* | AIC | BIC | Class probabilities* |
| --- | --- | --- | --- | --- | --- | --- | --- | --- | --- |
| traj_1 | 1 | NA | NA | NA | NA | NA | 50660.83 | 50696.04 | 1.000 |
| traj_2 | 0.966 | 0.970 | NA | NA | NA | 21.7 | 42987.27 | 43057.70 | 0.397 |
| traj_3 | 0.927 | 0.933 | 0.941 | NA | NA | 15.6 | 41109.46 | 41215.11 | 0.212 |
| traj_4 | 0.910 | 0.914 | 0.933 | 0.884 | NA | 14.2 | 40417.53 | 40558.39 | 0.143 |
| traj_5 | 0.824 | 0.904 | 0.873 | 0.951 | 0.878 | 14.5 | 40166.50 | 40356.66 | 0.064 |

Note: *Represents only presenting the minimum value among multiple values


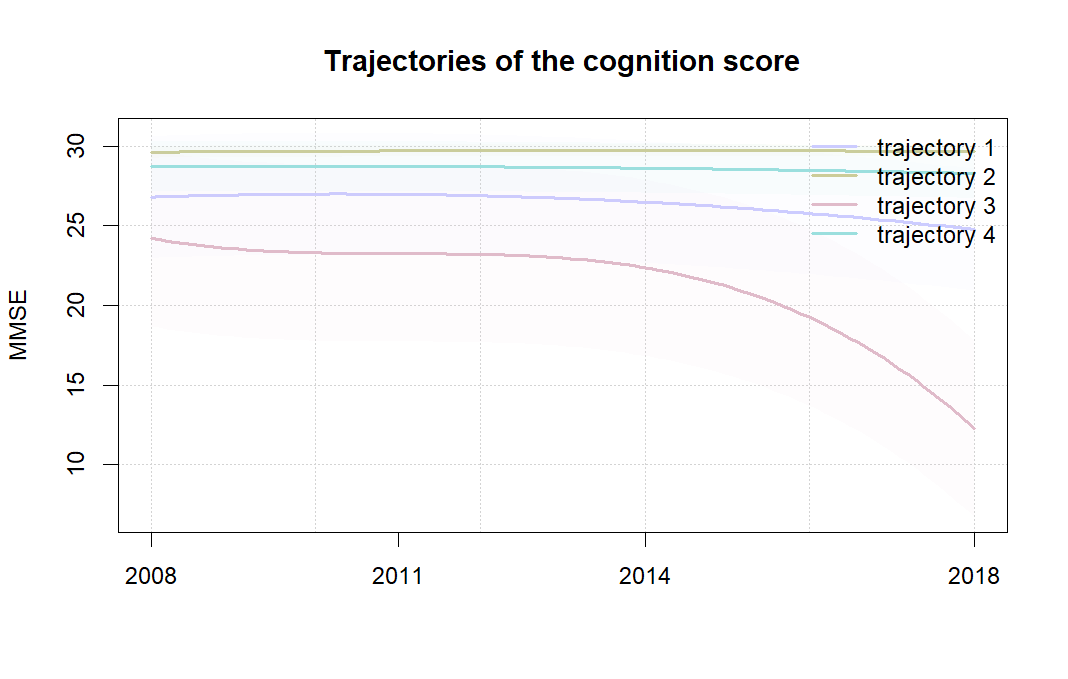


**Supplementary Figure 3.** Trajectories of the MMSE scores.

## Association Between Cognitive Trajectories and years of education and Diet

**Supplementary Table 3.** Association between cognitive trajectories and years of education and diet

|  | Stable Decline / High Stability | | | | Rapid Decline / High Stability | | | |
| --- | --- | --- | --- | --- | --- | --- | --- | --- |
|  | *β* | *Z* | *OR (95%CI)* | *P* | *β* | *Z* | *OR (95%CI)* | *P* |
| FV_20_8 |  |  |  |  |  |  |  |  |
| Model1 | -0.121 | -6.062 | 0.886(0.852~0.921) | <0.001 | -0.121 | -6.239 | 0.842(0.797~0.889) | <0.001 |
| Model2 | -0.082 | -3.776 | 0.922(0.883~0.961) | < 0.001 | -0.082 | -3.814 | 0.884(0.83~0.942) | <0.001 |
| Model3 | -0.079 | -3.581 | 0.924(0.885~0.965) | < 0.001 | -0.079 | -3.551 | 0.889(0.834~0.949) | <0.001 |
| PDI |  |  |  |  |  |  |  |  |
| Model1 | -0.378 | -0.946 | 0.686(0.313~1.499) | 0.344 | -0.378 | 0.183 | 1.1(0.397~3.044) | 0.855 |
| Model2 | 0.008 | 1.012 | 1.008(0.992~1.025) | 0.31167 | 0.008 | -0.632 | 0.992(0.969~1.016) | 0.527 |
| Model3 | 0.011 | 1.313 | 1.011(0.994~1.028) | 0.18912 | 0.011 | -0.412 | 0.995(0.971~1.019) | 0.680 |
| hPDI |  |  |  |  |  |  |  |  |
| Model1 | -0.012 | -1.459 | 0.988(0.971~1.004) | 0.145 | -0.012 | -2.179 | 0.976(0.954~0.998) | 0.029 |
| Model2 | <0.001 | 0.041 | 1.000(0.983~1.018) | 0.967 | <0.001 | -0.060 | 0.999(0.974~1.025) | 0.952 |
| Model3 | 0.003 | 0.278 | 1.003(0.984~1.021) | 0.781 | 0.003 | 0.171 | 1.002(0.976~1.029) | 0.864 |
| uPDI |  |  |  |  |  |  |  |  |
| Model1 | 0.035 | 3.900 | 1.036(1.018~1.054) | <0.001 | 0.035 | 1.763 | 1.021(0.998~1.046) | 0.078 |
| Model2 | 0.034 | 3.507 | 1.034(1.015~1.054) | < 0.001 | 0.034 | 1.950 | 1.027(1~1.056) | 0.051 |
| Model3 | 0.034 | 3.496 | 1.034(1.015~1.054) | < 0.001 | 0.034 | 1.895 | 1.027(0.999~1.055) | 0.058 |
| Education |  |  |  |  |  |  |  |  |
| Model1 | -0.227 | -13.106 | 0.797(0.770~0.824) | < 0.001 | -0.227 | -10.979 | 0.720(0.679~0.763) | <0.001 |
| Model2 | -0.166 | -8.755 | 0.847(0.817~0.879) | <0.001 | -0.166 | -6.255 | 0.817(0.767~0.87) | <0.001 |
| Model3 | -0.164 | -8.585 | 0.848(0.817~0.881) | <0.001 | -0.164 | -6.140 | 0.818(0.768~0.872) | < 0.001 |

## Network analysis results

| 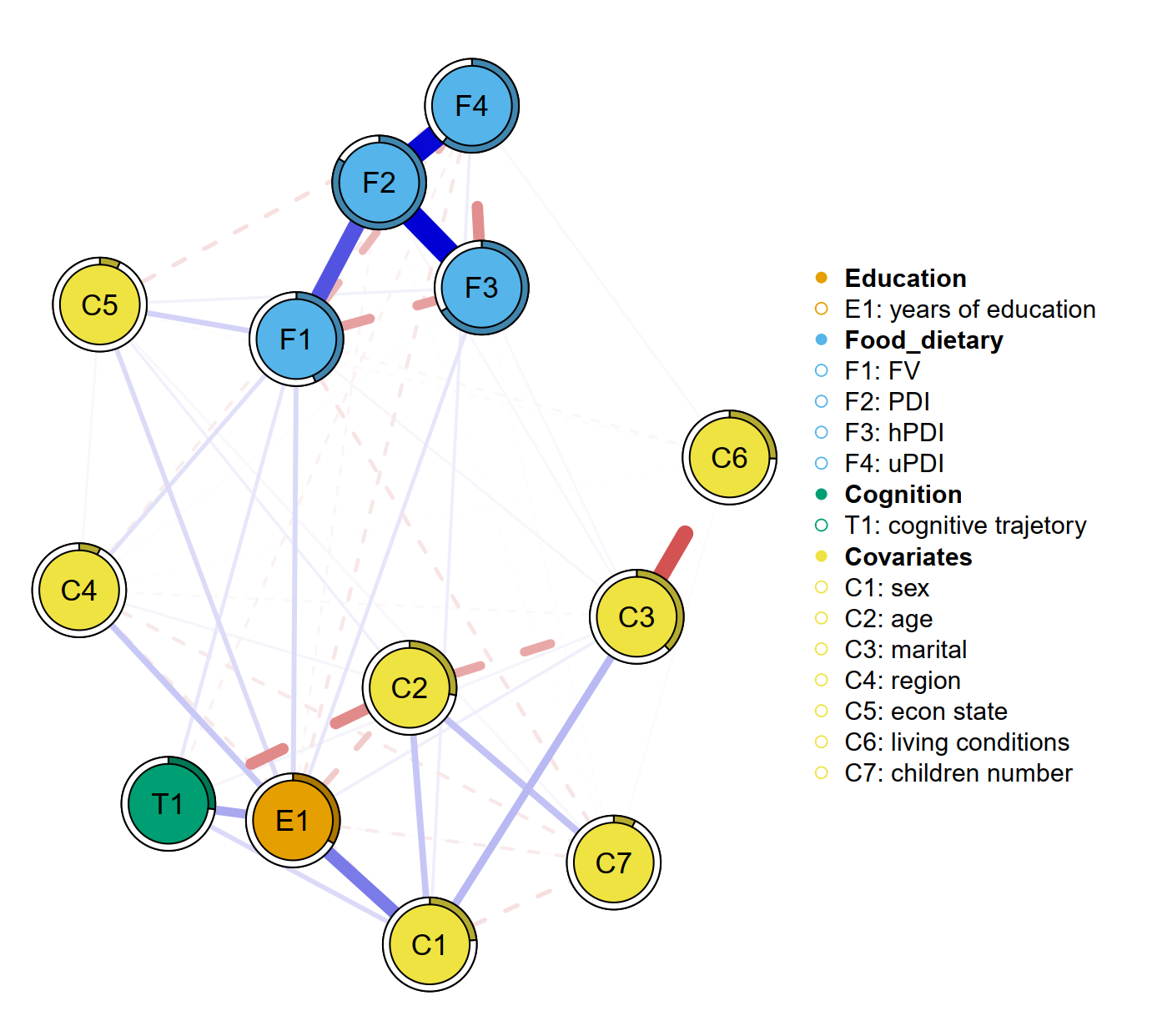 | 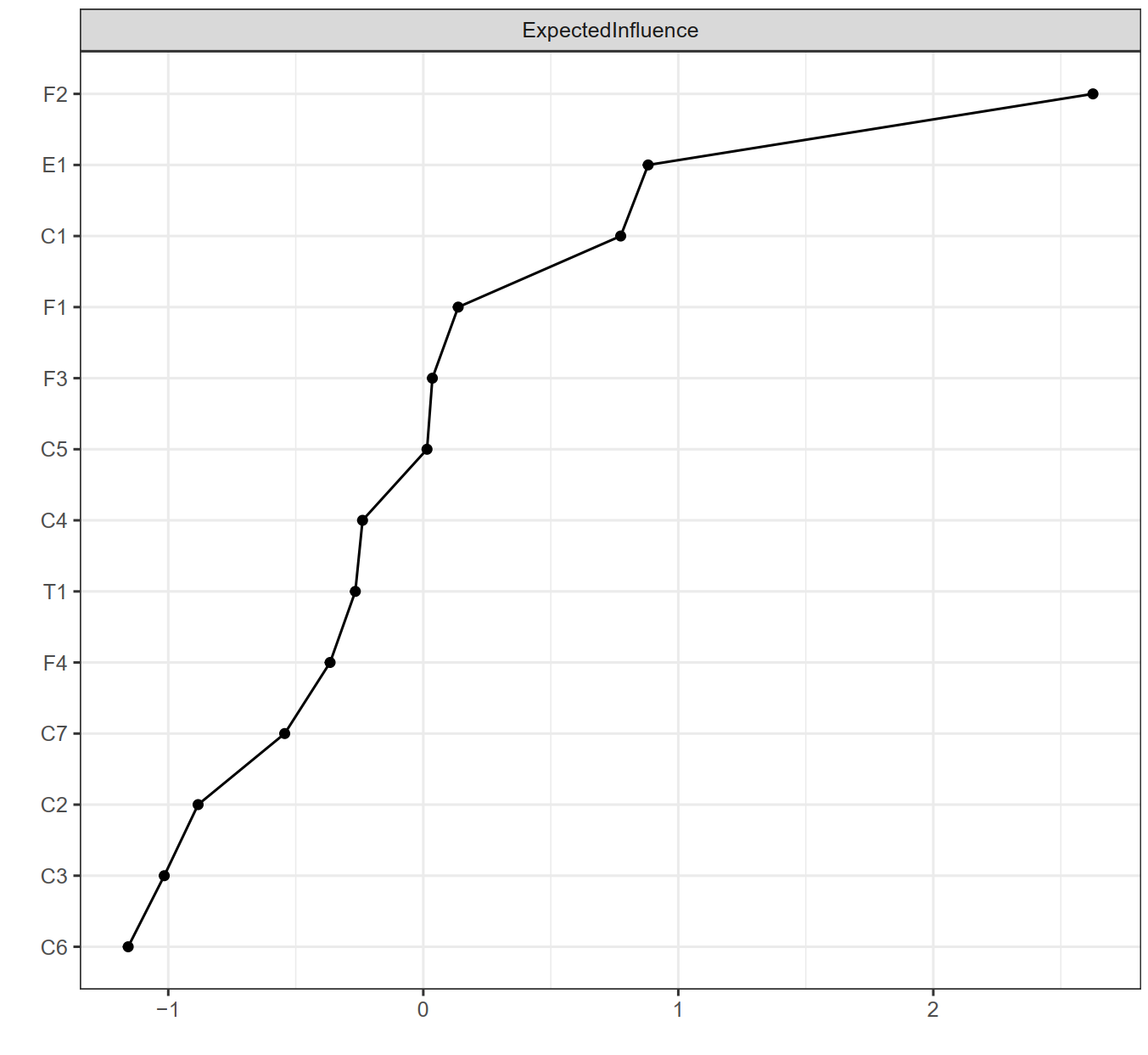 |
| --- | --- |

**Supplementary Figure 4.** Network sensitivity analysis including age, gender, region, marital, econ state, living conditions and children number.

| 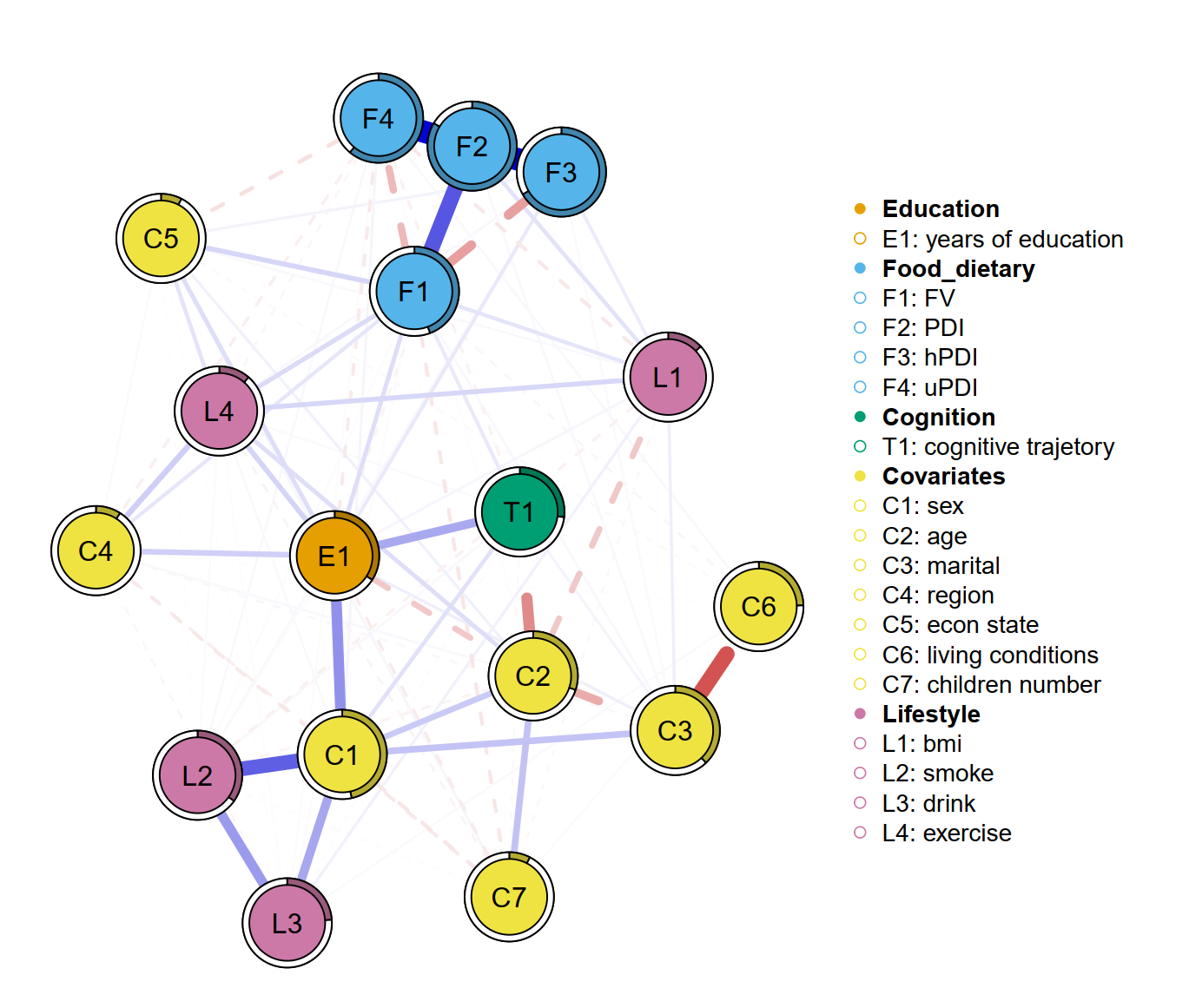 | 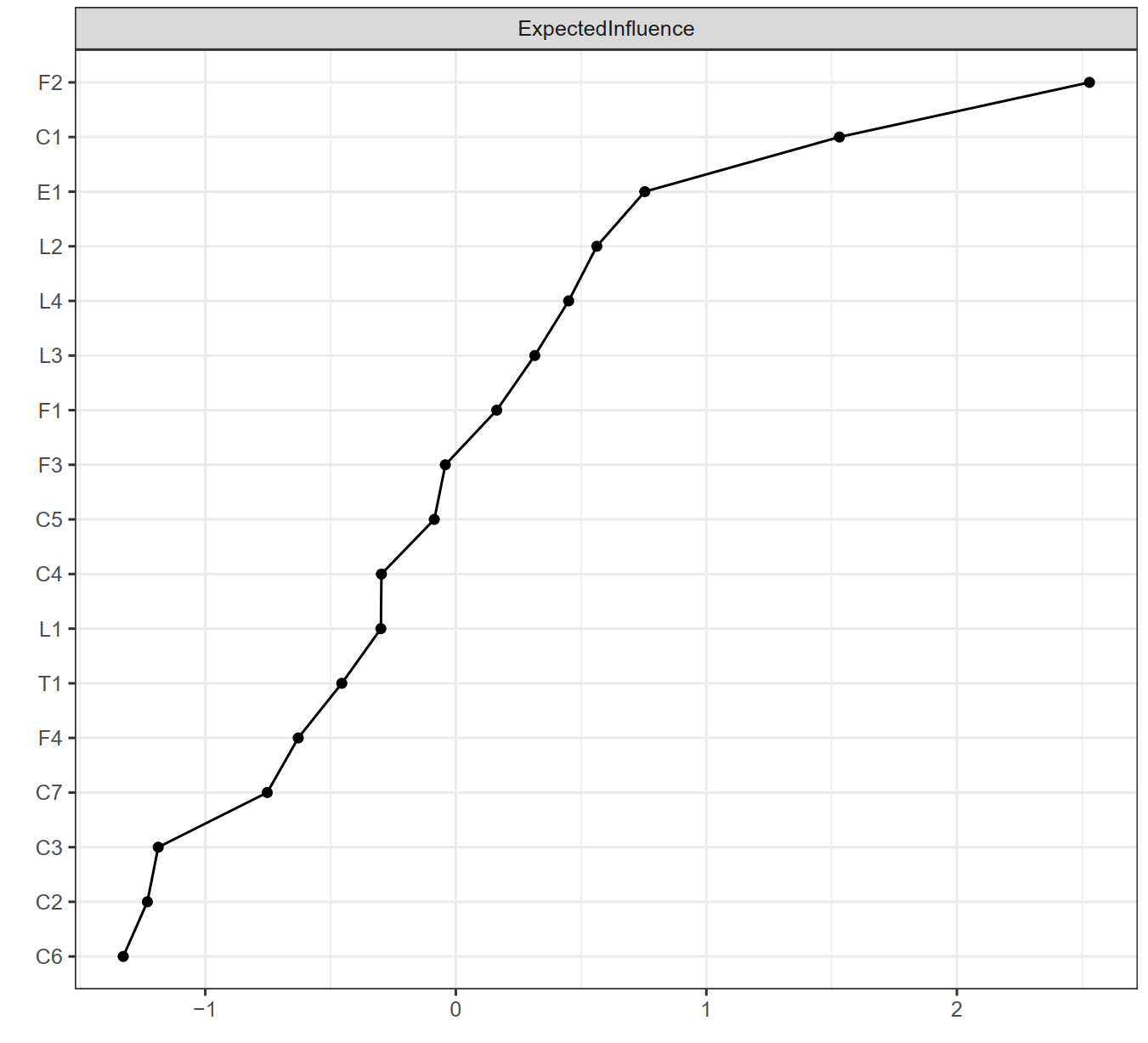 |
| --- | --- |

**Supplementary Figure 5.** Network sensitivity analysis including age, gender, region, marital, econ state, living conditions, children number, smoke, drink, exercise and BMI.


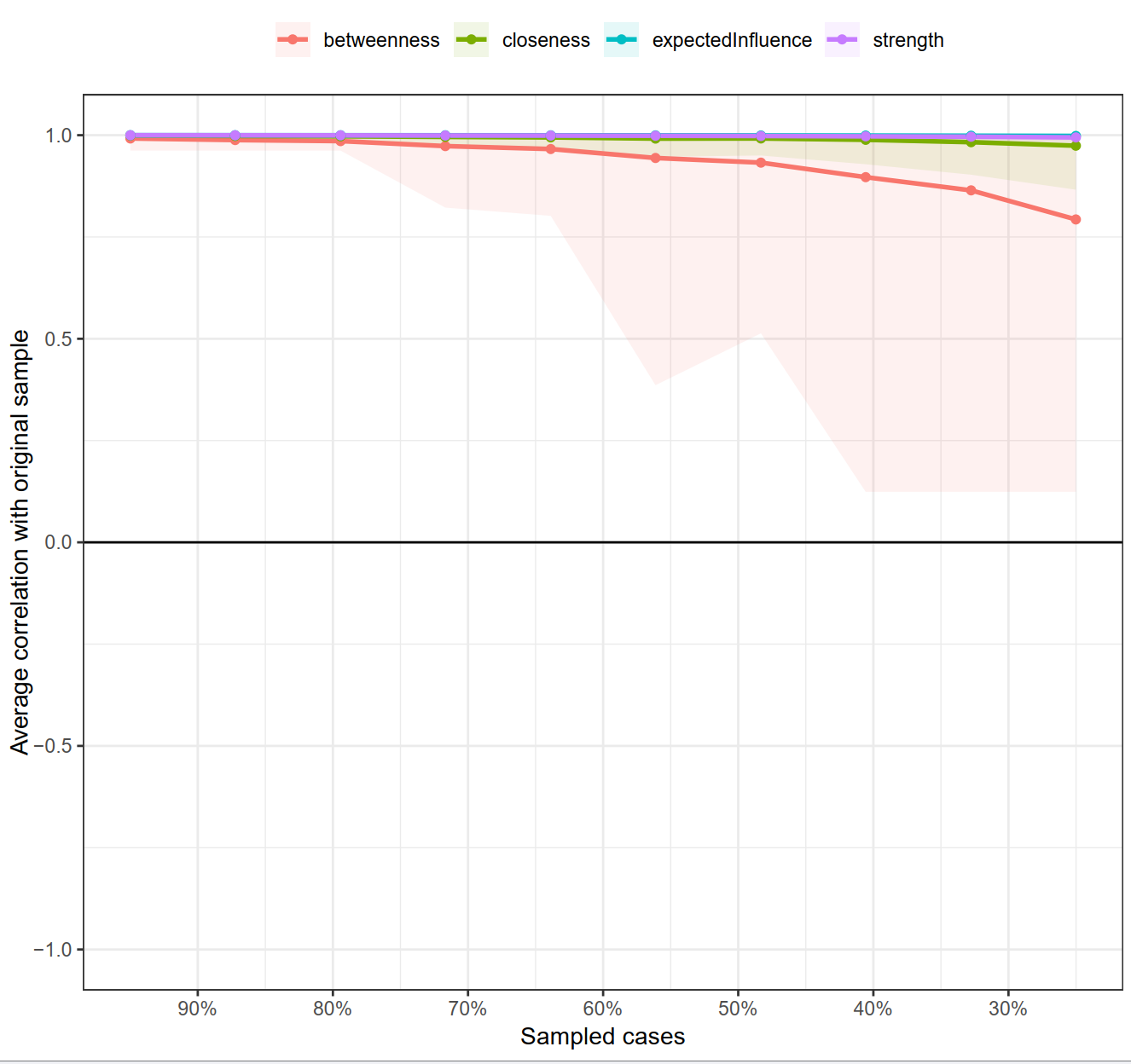


**Supplementary Figure 6.** Bootstrapped stability of primary network analysis.


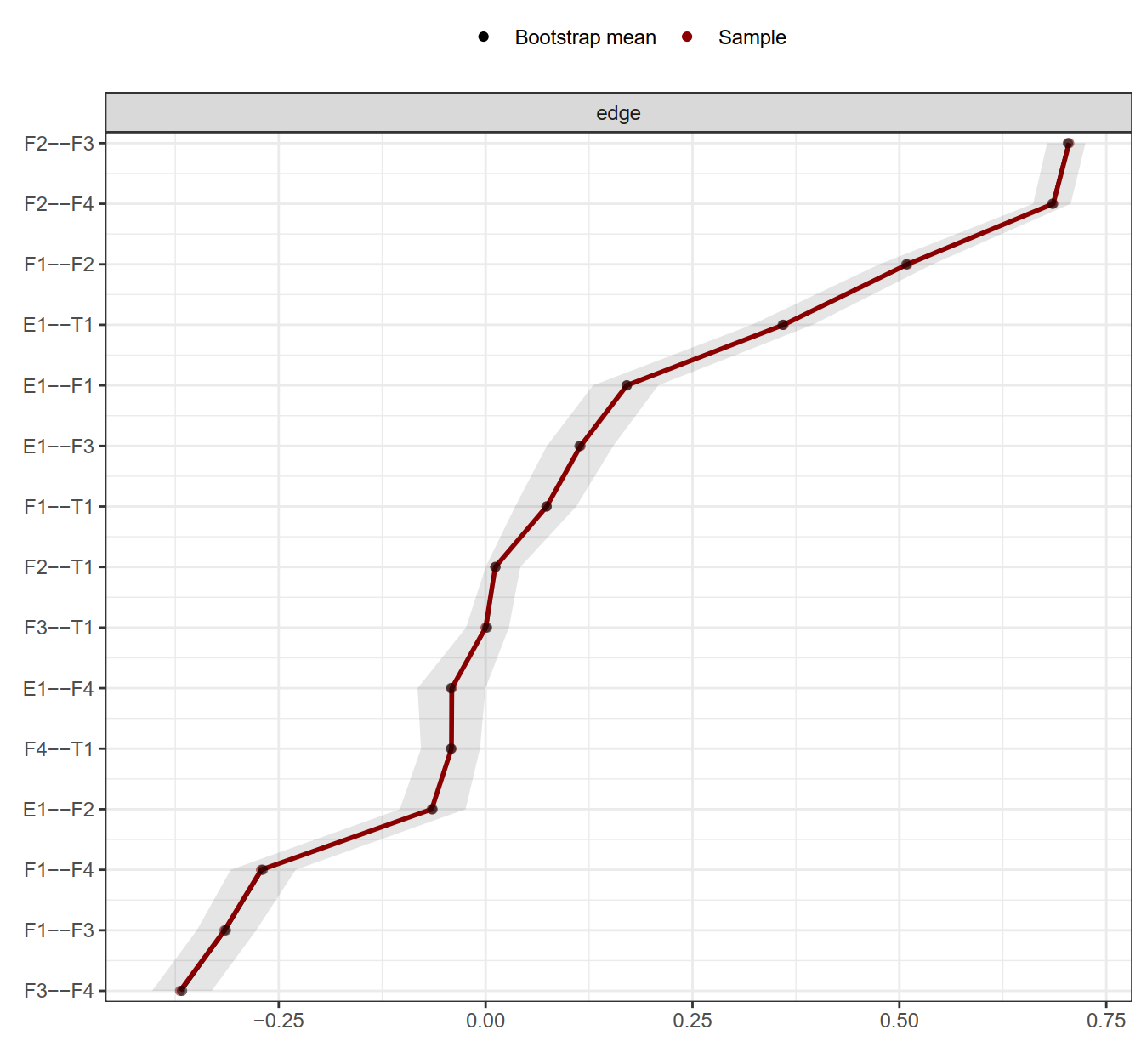


**Supplementary Figure 7.** Bootstrapped confidence intervals of edge weights of primary network analysis.


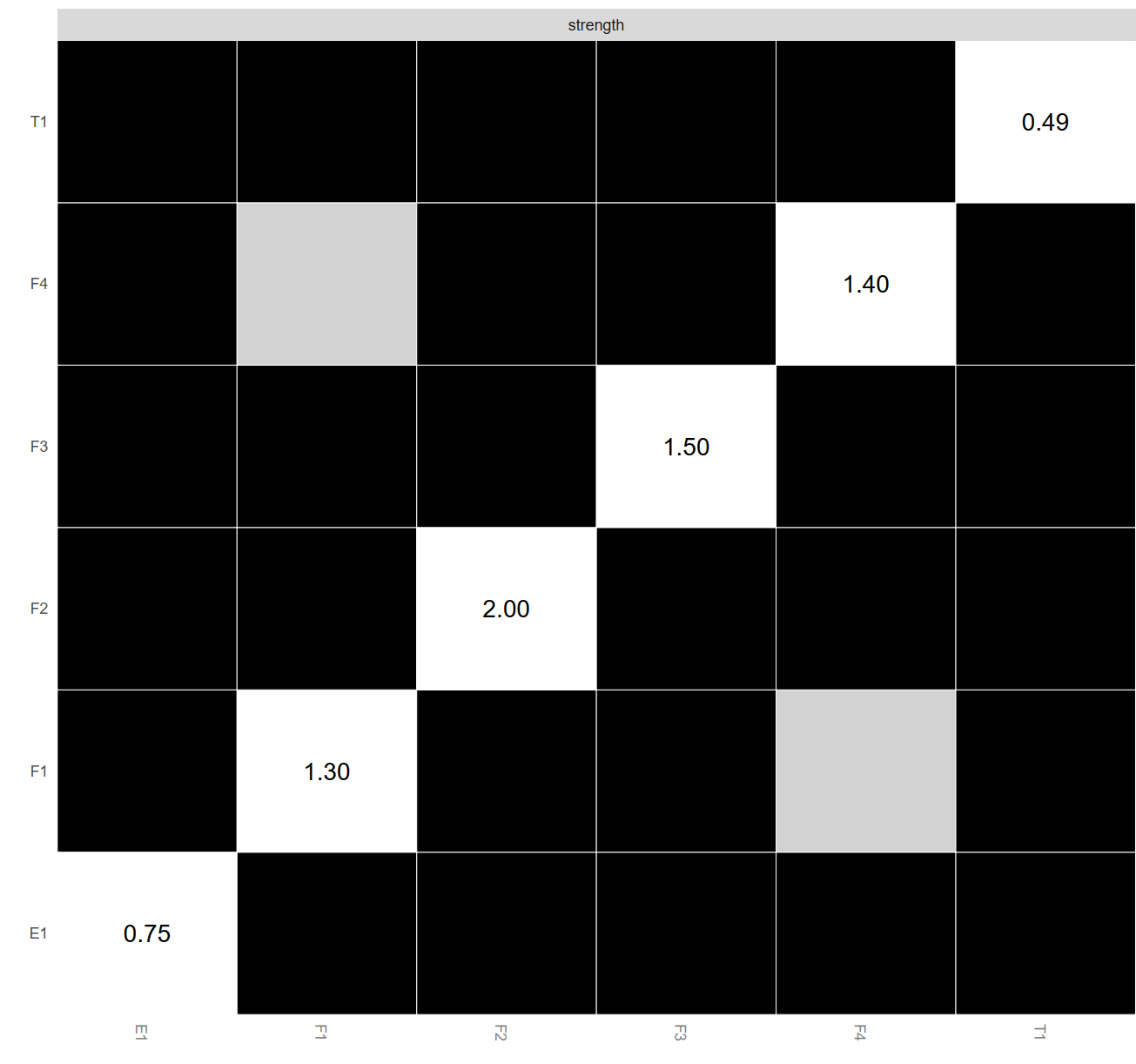


**Supplementary Figure 8.** Bootstrapped test for confidence intervals of EI of primary network analysis

Note: Grey boxes indicate nodes that do not differ significantly from one-another (p <0.05) and black boxes represent nodes that do differ significantly from one-another.


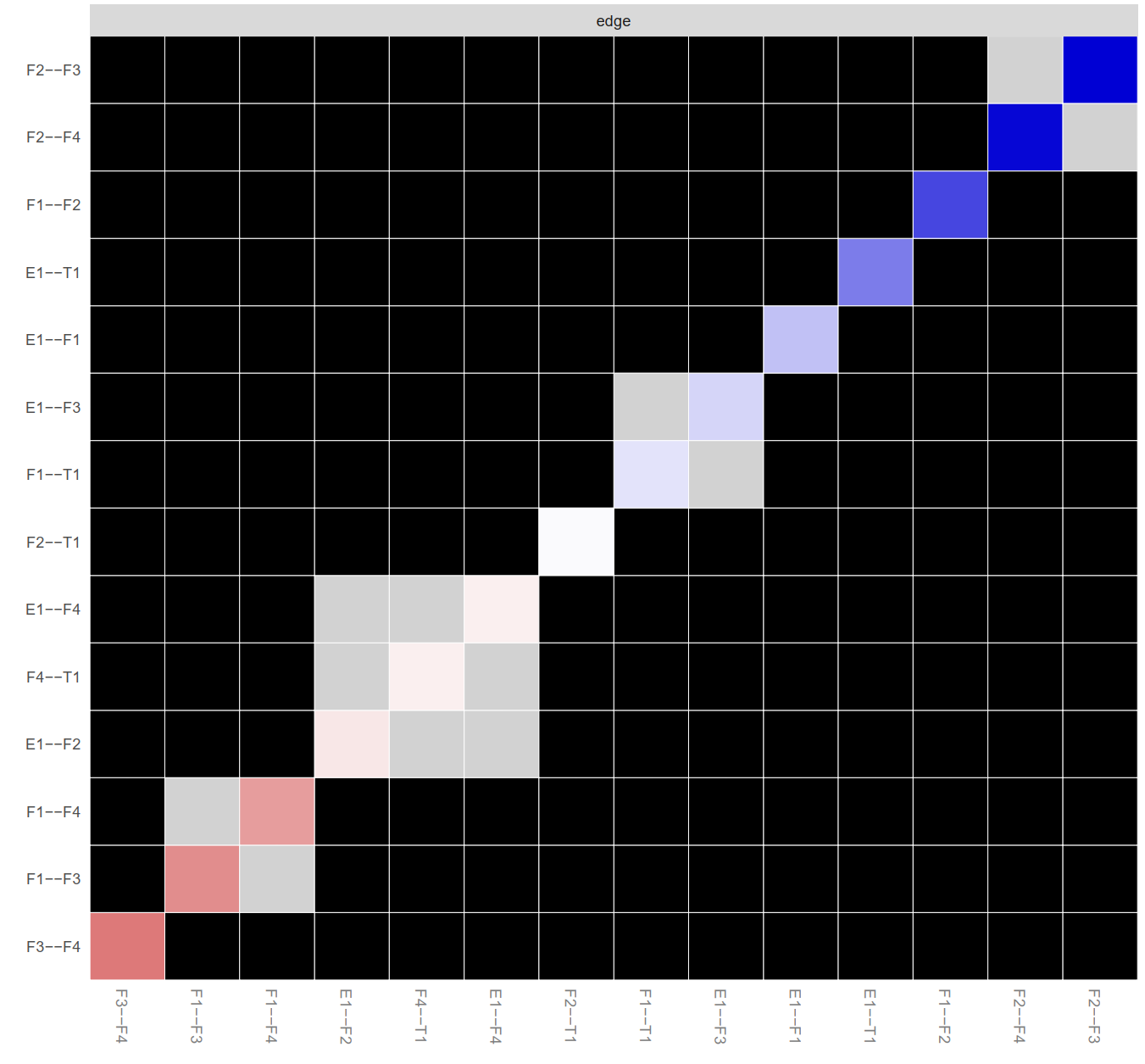


**Supplementary Figure 9.** Bootstrapped difference test for edge weight of primary network analysis.

## Longitudinal association Between Education, Diet and Cognitive


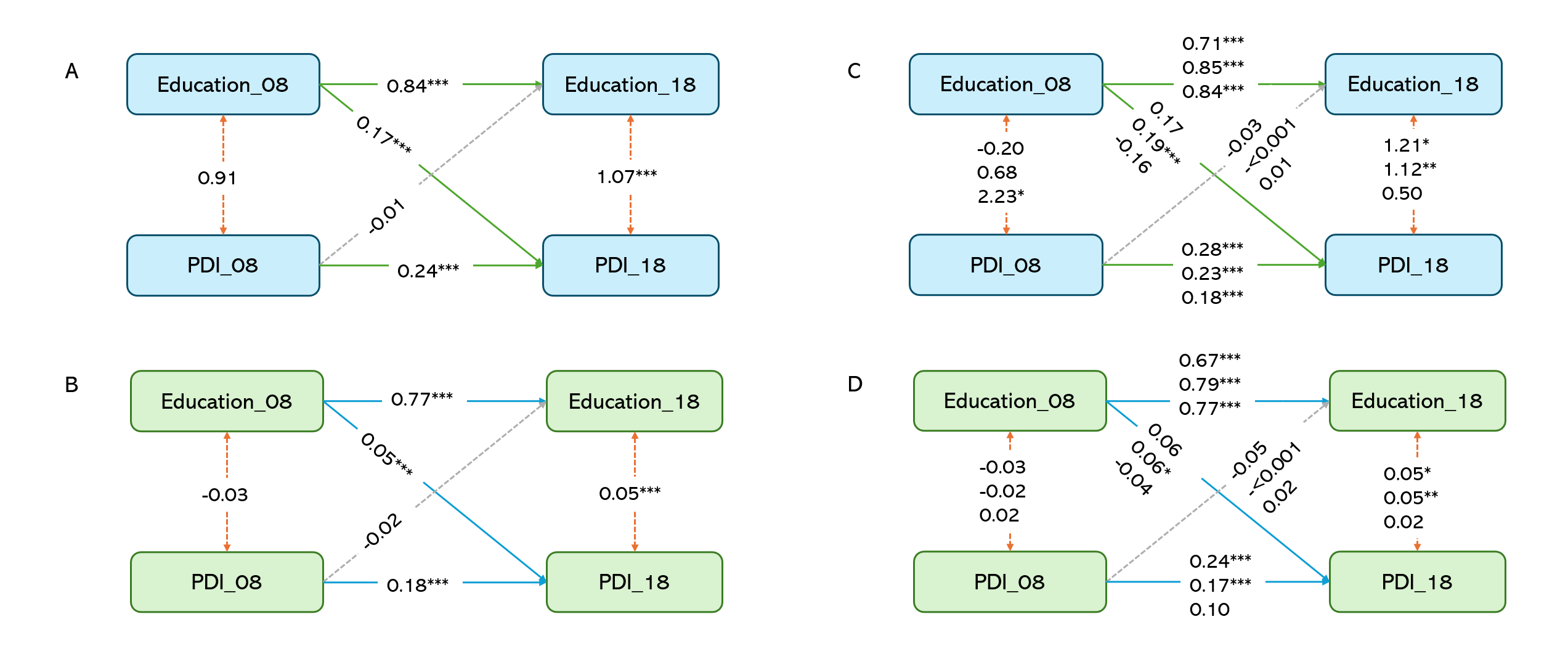


**Supplementary Figure 10.** Cross lagged model diagram between education and PDI.


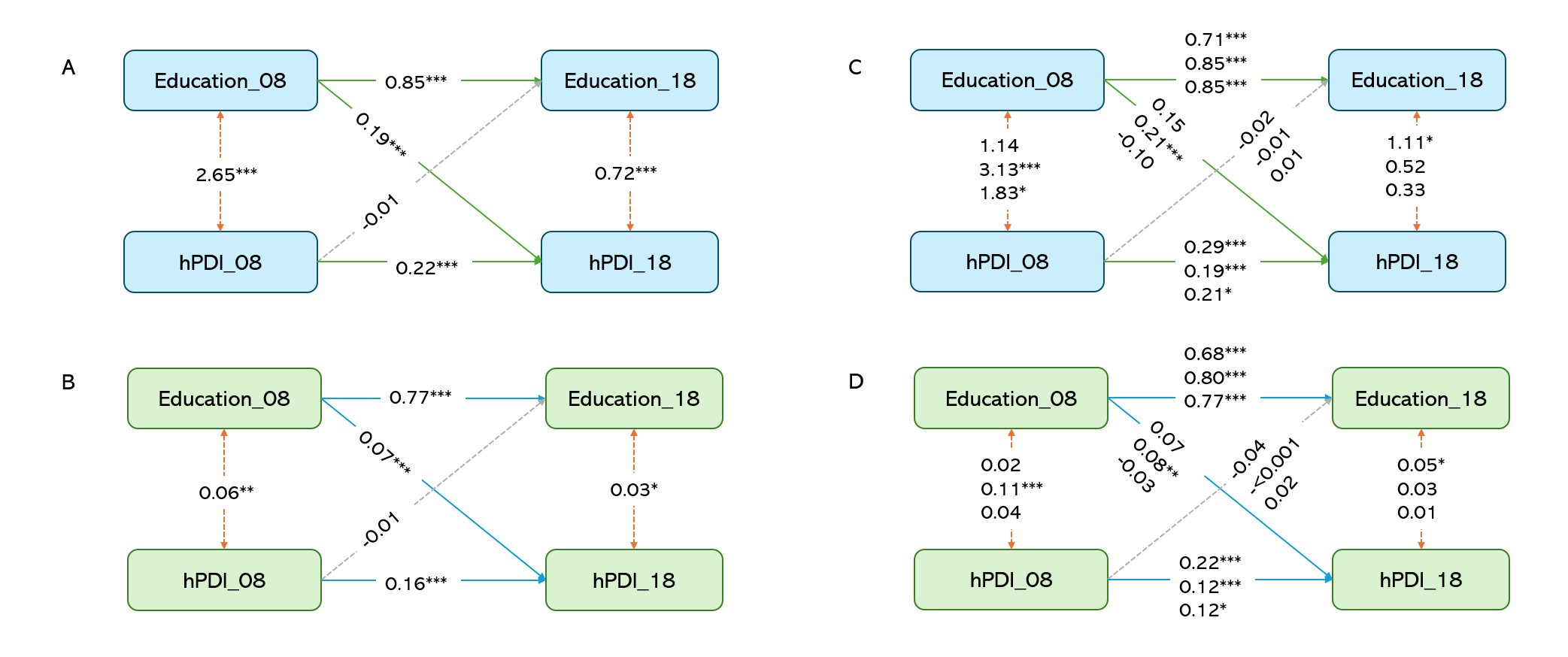


**Supplementary Figure 11.** Cross lagged model diagram between education and hPDI.


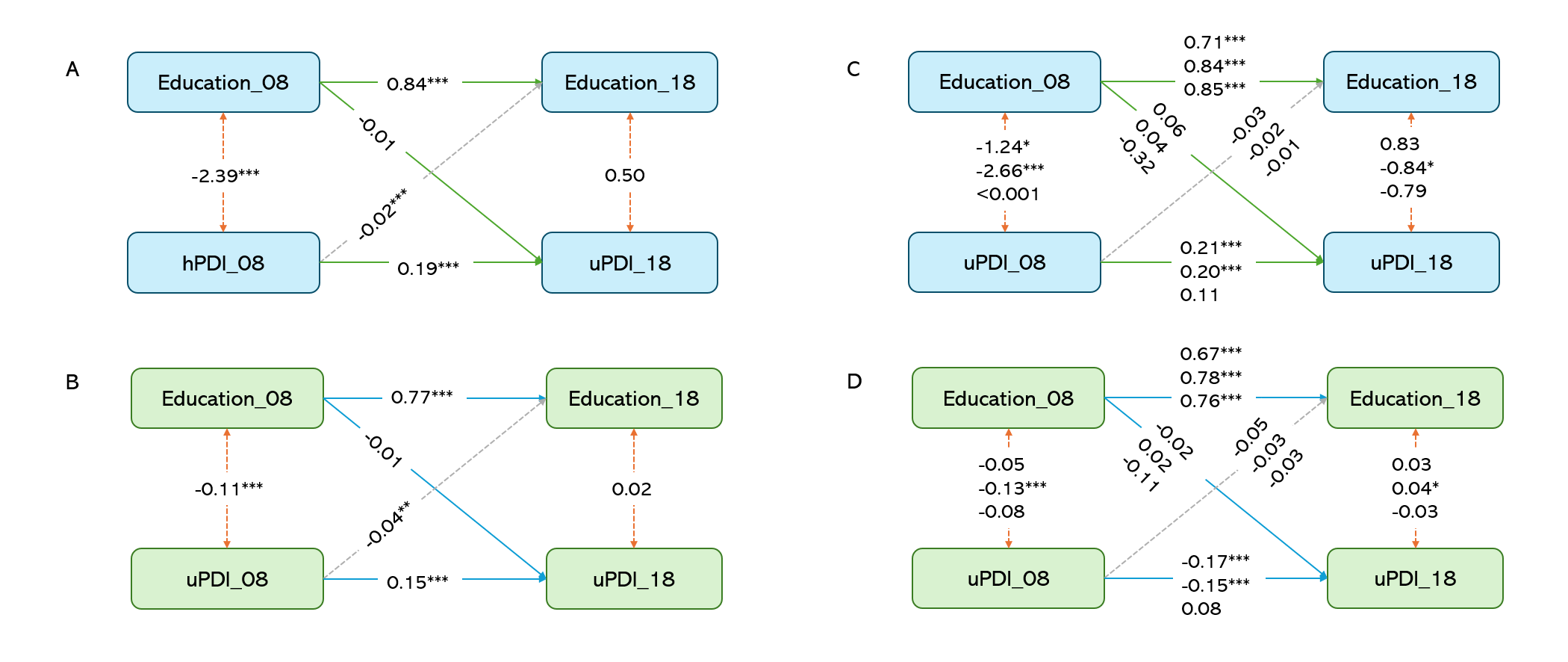


**Supplementary Figure 12.** Cross lagged model diagram between education and uPDI.


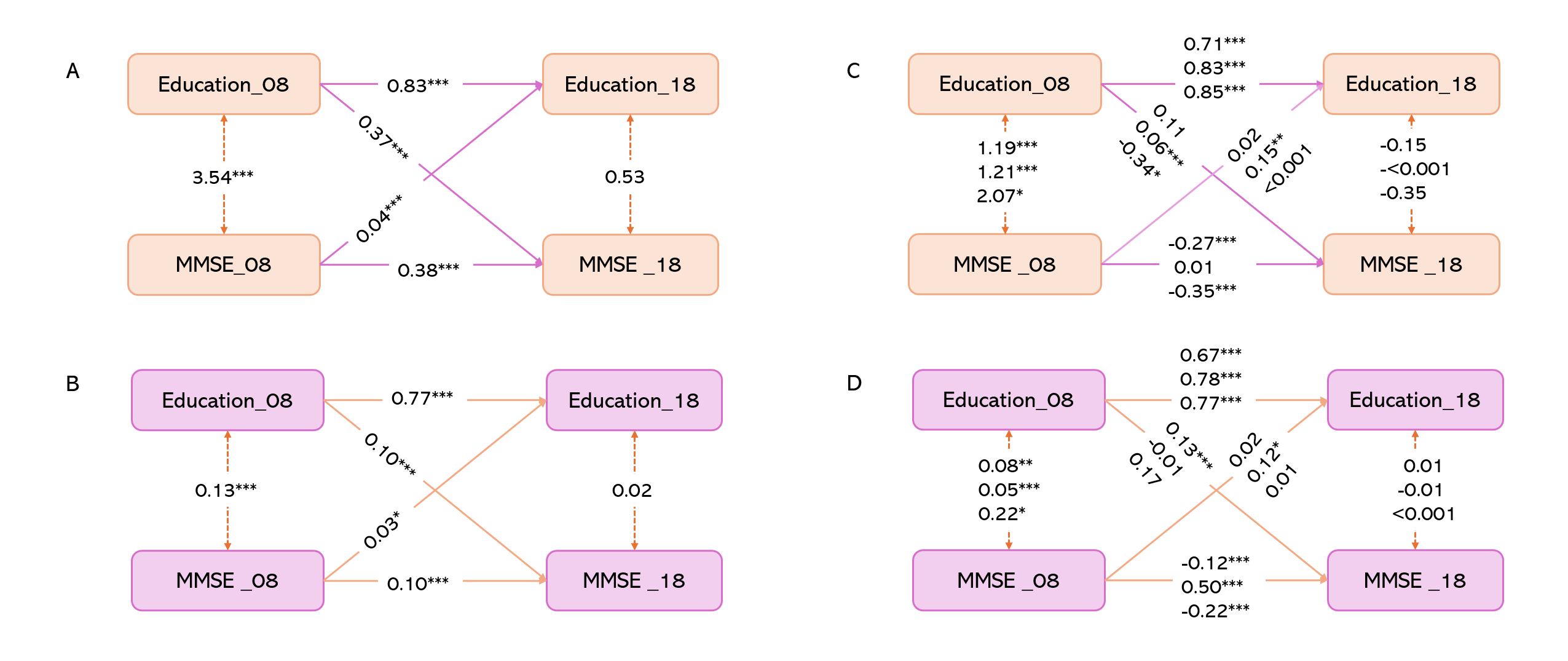


**Supplementary Figure 13.** Cross lagged model diagram between education and MMSE.
